# Differences in patient-physician communication between the emergency department and other departments in a hospital setting in Taiwan

**DOI:** 10.1186/s12913-023-10311-2

**Published:** 2023-11-20

**Authors:** Yi-Fen Wang, Ya-Hui Lee, Chen-Wei Lee, Yu-Ze Shih, Yi-Kung Lee

**Affiliations:** 1 Department of Senior Citizen Services, National Tainan Junior College of Nursing, Tainan, Taiwan; 2 Department of Adult & Continuing Education, National Chung Cheng University, Chiayi, Taiwan; 3 Emergency Department, Dalin Tzu Chi Hospital, Buddhist Tzu Chi Medical Foundation, No. 2, Minsheng Rd., Dalin Township, Chiayi County 622 Taiwan; 4 School of Post-Baccalaureate Chinese Medicine, Tzu Chi University, Hualien, Taiwan; 5 School of Medicine, Tzu Chi University, Hualien, Taiwan

**Keywords:** Emergency department, Patient-physician communication, Continuing medical education

## Abstract

**Background:**

Communication fosters trust and understanding between patients and physicians, and specific communication steps help to build relationships. Communication in the emergency department may be different from that in other departments due to differences in medical purposes and treatments. However, the characteristics of communication in the clinical settings of various departments have not been explored nor compared.

**Objectives:**

This study aimed to construct the steps in patient-physician communication based on the Roter Communication Model and compare communication performance between the emergency department and three other clinical settings—internal medicine, surgery, and family medicine departments.

**Methods:**

Both qualitative and quantitative approaches were adopted. First, in-depth interviews were used to analyze clinical communication steps and meanings. Then, a quantitative questionnaire was designed based on the interview results to investigate differences in communication between the emergency department and the other three departments. Qualitative and quantitative data were analyzed from 20 interviews and 98 valid questionnaires.

**Results:**

Patient-physician communication consists of four steps and ten factors. The four steps—greeting and data gathering, patient education and counseling, facilitation and patient activation, and building a relationship—had significant progressive effects. Patient education and counseling had an additional significant effect on building a relationship. The emergency department performed less well in the facilitation and patient activation, building a relationship step and the evaluation method, enhancement method, and attitude factors than the other departments.

**Conclusions:**

To improve the quality of patient-physician communication in the emergency department, physicians should strengthen the steps of facilitation and patient activation to encourage patients’ active engagement in their health care.

**Supplementary Information:**

The online version contains supplementary material available at 10.1186/s12913-023-10311-2.

## Background

Good communication is the cornerstone of building the patient-physician relationship [1, 2] and positively correlates with the patient's trust and satisfaction with the physician [3]. The average patient has only about 22 s to give an initial account of their complaint to the outpatient physician before the latter takes over the conversation [4]. Emergency departments are often busy and congested, and labor shortages mean that medical staff must make judgments in very short, fragmented periods [5, 6] so that patients have only about 14 s to speak. Only 16% of patients are even asked if they understand the medical information or have any questions [7]. This suggests that the communication process is mostly physician-driven, and medical professionals lack the time to properly listen to the patient. Moreover, most medical training does not cover communication skills, and physicians in emergency medicine are somewhat unmotivated to improve their communication skills because of negative factors, such as high stress, heavy workload, and overtime [8]. This emphasizes the importance of taking practical steps to communicate in the emergency department and the appropriate modes of medical communication.

The Roter Interaction Analysis System (RIAS) is often used to study doctor–patient communication behaviors. According to a previous study, patient education and counseling (48.9%) and building a relationship (32.5%) are the most typical incentives for communication in general outpatient clinics, far more than the need for patient facilitation and activation (13%) and data gathering (3.4%) [9]. In addition, the communication processes are led by physicians, who tend to emphasize biomedical information more than patients' life stories and psychological status, and the medical team speaks more (73%) than the patients (23%) [10]. The central part of the communication is devoted to patient education and consultation (34%) and patient facilitation and activation (30%) than to building a relationship (21%) and data gathering (15%). Patient education and consultation are emphasized in all types of patient-physician communication contexts. However, the first task of the emergency physician is to relieve the patient's acute symptoms and provide specialty care. In contrast, relationship building, patient activation, and data collection are not likely to be prioritized in emergency settings. Although the RIAS model clearly defines patient-centered communication, it does not provide specific identified steps [11] for effective communication, so there are limitations in its applicability to patient-physician communication.

Good communication reinforces the patient's trust in the physician and guides the physician to employ supportive strategies that improve patient compliance and ensure better health outcomes. Previous studies identified the following components of successful communication between patients and physicians. The first consists of greeting and data gathering. Initial words such as a greeting or self-introduction, complemented by handshaking and eye contact, can decrease the patient's anxieties, create a positive impression [12–14], and increase the patient's willingness to self-report the reason for the visit. The second component consists of open-ended questions for gathering information and closed-ended questions for summarizing and validating the information received [1, 15], so that patients feel understood and listened to. The third component consists of the patients' expectations of physicians being caring, respectful, and empathetic [16, 17], as well as providing medical information and care to improve the patients' psychology or lifestyle. Nonverbal behaviors such as facial expressions, gaze, distance, voice, touch, and posture [3, 18, 19] may prompt patients to express their thoughts or fears willingly. Facilitating patient activation is also important. By discussing medical decisions together, physicians can understand the feasibility of patient compliance [20] and provide positive encouragement [2] to motivate positive patient activation, thereby building up a patient-physician relationship. A trusting relationship helps to increase satisfaction, resulting in fewer days in the hospital, lower readmission rates, and more effective care [21, 22].

In summary, communication between physicians and patients promotes interactions and builds positive relationships. The emergency department is unique in its mode of patient-physician interaction due to the purpose and atmosphere of the clinical setting; therefore, the focus of communication differs from that of other departments [23]. Although some studies have identified practical steps for communication [18], the interrelationship between the measures has not been further explored, nor has the application of the steps to comparisons between different specialties. Therefore, this study aimed to construct the steps of patient-physician communication and their interactions and compare the differences in communication between the emergency department and other clinical departments.

### Objectives

Patient-physician communication promotes trust and understanding between the participants, and specific behavioral steps can help to build relationships. However, communication behavior in the emergency department, which is affected by the purpose and atmosphere of treatment, is different from that in other medical departments, an aspect that has hitherto not been explored. This study aimed to examine the factors and relationships that influence patient-physician communication from the Taiwanese perspective and to further compare the differences between the emergency department and other departments. The objectives of the study were (a) to understand the factors influencing patient-physician communication behavior, (b) to explore the relationship between physician and patient communication behaviors, and (c) to compare the differences in patient-physician communication behavior between the emergency department and other departments.

## Methods

Before conducting this study, the institutional review board of the study hospital approved this study and confirmed that the protocol did not jeopardize the respondents' interests. A mixed method of qualitative and quantitative approaches was adopted. Firstly, a qualitative one-on-one interview was performed to summarize the physician–patient communication experience. Then, a quantitative questionnaire survey was used to analyze the influencing factors and physician–patient relationships. The research instrument was produced by consolidating the literature and the practical experience of the authors as well as expert validity examination.

### Qualitative data collection and analysis

In-depth interviews were conducted to collect qualitative data [24, 25] to understand patient-physician communication experiences from respondents' narratives, identify critical factors, and generalize clinical communication behaviors and meanings.

#### Participant recruitment

Purposive sampling was used to select regional hospitals with the highest percentage of older patients in Taiwan, where 21.69% of the population is over 65 years of age [26]. Internal, surgical, family medicine, and emergency department-attending physicians with at least one year of experience were the target professional population. Further snowball sampling was used to obtain reliable information by asking the respondents to recommend those with the same experience [27].

#### Research tools

The interview outline was used as a tool for the researcher to generate interview questions based on literature review findings and the study's purpose. These questions included: (a) How are patients interviewed to gather information about their condition? (b) How are patients asked to collect information about the disease? How is the disease and health counseling explained to patients? (c) How are good relationships established with patients? (d) How is patients' compliance with medical advice assessed? How are the ways to improve patients' compliance assessed? (see Additional file 1).

#### Procedure

After review by the hospital's Research Ethics Committee, the researcher contacted eligible respondents individually between January and March 2021 to obtain each respondent's consent and sign an informed consent form. Qualitative method and research ethics-qualified researchers conducted a single interview with each respondent through communication software (Zoom). However, given physicians' busy schedules, approximately 15–25% of attending physicians at the regional hospitals eligible for the study were asked to participate in the interviews.

#### Data analysis

After the interviews were completed, the audio files were transcribed verbatim. The data were analyzed using the constant comparative method, with the concepts compiled into categories [28]. Finally, themes were identified to summarize the essential aspects and factors of patient-physician communication (see Table 1). In the analysis process, the researcher adopted triangulation [29], in which two interviewers and three members of the research team, as well as the respondents, were invited to interactively review and compare the analyzed data to strengthen the credibility and validity of this study.

**Table 1:** Example of the data analysis

**Themes**	**Sub-themes**	**Meaning units**	**Summary of meanings**
Building a relationship	Attitude	Mutual trust between the parties	Communication is only effective when trust is achieved with the patient
			If you don't have trust at the beginning, you can't listen to anything you say
			If the patient trusts me and I trust him to listen to me, the communication will be effective
		Showing sincerity to help	Demonstrate concern and sincerity in trying to help patients
			Let the patient feel that you can help them solve their problem

### Quantitative data collection and analysis

The quantitative questionnaire was designed to allow attending physicians to self-assess their conformity with the expected "communication behaviors" to show the behavioral trends and their influences on the relationship [30].

#### Participant recruitment

Using the aforementioned regional hospitals as the scope of this study, a random sample of 30 doctors from each of the four disciplines of internal, surgery, and family medicine and emergency departments were surveyed following convenience sampling of 120 attending physicians. This sampling aligns with Gay's recommendation that each group have a sample size of 30 when conducting relevant analyses or comparative studies [31].

#### Research tools

The questionnaire was developed with 46 questions, such as "Do you greet and introduce yourself to patients first?" and "Do you ask patients about their personal information and past medical history?" Response options were categorized according to frequency of use: mostly yes, depending on the situation, and mostly no (see Additional file 2).

#### Procedure

After removing the samples that were not tested or invalid, 98 physicians ultimately remained (see Table 2), 24 each from the emergency and family medicine departments and 25 each from the internal and surgery departments. To compensate for the sample size which was below 30 for each department, this study adopted the "Structural Equation-Individual Parameter Checking Power Analysis," which first calculated the central parameters, confirmed the non-central parameters, and precisely estimated the π value by interpolation. To ensure the inference validity, the level of significant differences was set at *p* < 0.05 and the power was set at > 0.8, alongside the effect sizes and the difference between the median of the emergency department and the median of the whole sample in the analysis of variance with the other departments.

**Table 2:** Sample descriptive statistics

**Variable**	**Characteristic**	**Frequency**	**Percent**	**a Emergency**	**b** **Family medicine**	**c Internal**	**d Surgery**	**Chi-Squared Test**	
**Depart-ment**	a Emergency	24	24.49						
	b Family medicine	24	24.49						
	c Internal	25	25.51						
	d Surgery	25	25.51						
**Gender**	a Male	86	87.76	23	16	22	25	14.88	**
	b Female	12	12.24	1	8	3	0		
**service tenure**	a Less than 1 year	13	13.27	3	5	3	2	8.38	
	b From 1–4 years	7	7.14	1	2	2	2		
	c From 4–7 years	15	15.31	5	5	4	1		
	d From 7–10 years	5	5.10	2	0	1	2		
	e More than 10 years	58	59.18	13	12	15	18		
**Variable**	**Characteristic**	**Mean**	**Std. Deviation**	**a Emergency**	**b Family medicine**	**c Internal**	**d Surgery**	**F test**	
**Age**	28 ~ 71	43.98	9.75	43.88	40.58	43.40	48.08	2.53	

#### Data analysis

LISREL 8.51 and SPSS 21 software were used for an overall pathway analysis and variance. Validity and effectiveness parameters were analyzed using a general linear model. The departments were found to show different behaviors at each stage of the patient-physician communication model. Therefore, this study used pathway analysis to infer the patterns of patient-physician communication in Taiwan and whether there were different influence pathways for behaviors, attitudes, and displays of professionalism. In addition, pathway analysis was used to determine whether there were differences between hospital departments in each patient-physician communication stage.

## Results

In this study, 20 physicians were interviewed; five from every of the internal, surgery, family medicine, and emergency departments. Each interview lasted approximately 40 to 60 min. The physicians included 15 males and 5 females aged 33–49 years and had 1–18 years of service. The results analyzed four themes as steps in the patient-physician communication process: greeting and data gathering, patient education and counseling, facilitation and patient activation; and building a relationship. As well as 10 subthemes were analyzed as influencing factors: the starting sentence, the contents inquired about, inquiry methods, topics in patient education, description methods, evaluation method, enhancement method, behaviors, attitudes, and display of professionalism.

According to the descriptive statistics (see Table 3), the mean of the components of patient-physician communication ranged from 2.736 to 2.922, with scores tending to be slightly high, but were normally distributed (skewness between 0.489 and 7.486 <|10| and kurtosis between -0.801 and -2.925 <|3|). The correlation coefficients of the relationships between the three steps, greeting and data gathering, patient education and counseling, and facilitation and patient activation, ranged from 0.436 to 0.666, indicating moderate levels of association, and, therefore, a certain degree of influence. The correlation coefficients between the above three steps and building a relationship ranged from 0.335 to 0.667, indicating moderate associations, but the correlation coefficient of the relationship between greeting and data gathering and display of professionalism was the lowest at 0.145. Therefore, greeting and data gathering did not seem to have a direct effect on the display of professionalism.

**Table 3:** Statistical table describing each step of patient-physician communication

**patient-doctor communication Step**	**DG**	**EC**	**FA**	**BR (Overall)**	**BR1 (Behavior)**	**BR2 (Attitude)**	**BR3 (Displaying Professionalism)**
**DG, Greeting and Data Gathering**	1.000						
**EC, Patient Education and Counseling**	0.506	1.000					
**FA, Facilitation and Patient Activation**	0.436	0.666	1.000				
**BR, Building a Relationship (Overall)**	0.375	0.545	0.560	1.000			
**BR1, Building a Relationship (Behavior)**	0.507	0.564	0.346	0.628	1.000		
**BR2, Building a Relationship (Attitude)**	0.399	0.573	0.667	0.857	0.505	1.000	
**BR3, Building a Relationship (Displaying Professionalism)**	0.145	0.335	0.434	0.617	0.205	0.547	1.000
**Average**	2.735	2.781	2.787	2.860	2.852	2.850	2.922
**Standard deviation**	0.184	0.212	0.280	0.217	0.227	0.255	0.248
**Skewness coefficient**	-0.801	-1.218	-1.223	-2.030	-1.502	-1.992	-2.925
**Kurtosis coefficient**	1.515	1.436	0.489	4.000	1.701	3.583	7.486

To understand the degree of influence of patient-physician communication at each step as well as to improve the rigor of inference, this study conducted a path analysis by calculating the Δχ_MFF_^2^ -1 value of the focal path parameter and estimating the non-centrality parameter for power analysis at the model degree of freedom of 1. The gender ratio was biased toward male doctors (87.76%; χ^2^ = 14.88, *p* < 0.05) due to hospital staffing. However, there was no significant mismatch in age (*F* = 2.53, *p* > 0.05) and length of service (χ^2^ = 8.38, *p* > 0.05). Therefore, the samples were representative of the four departments in terms of patient-physician communication.

**Fig. 1:**
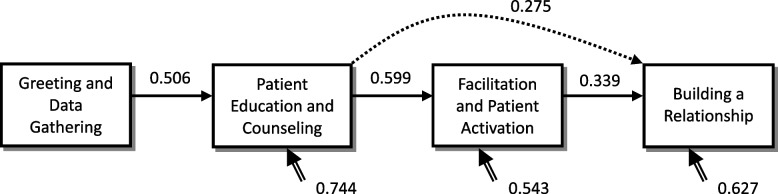
The four-step impact model of patient-physician communication (overall model) Real-line arrows, gamma coefficients (*p*  < 0.05, power > 0.8); Dashed arrows, gamma coefficient (*p*  < 0.05, power < 0.8); Double-stranded arrows, psi coefficients

**Fig. 2:**
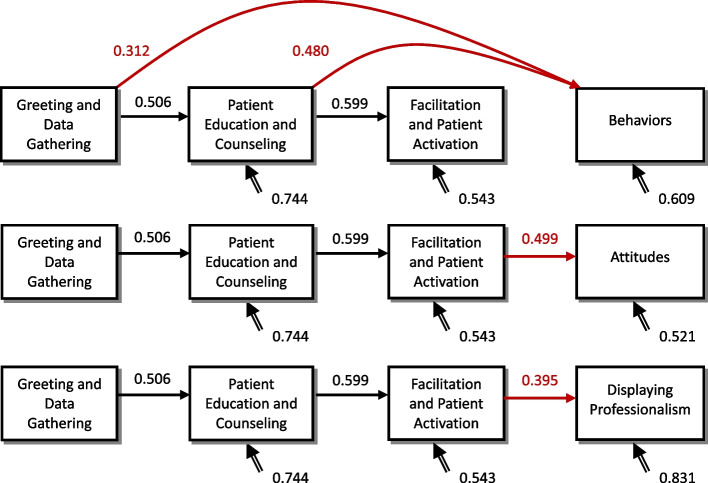
Diagram of the four-step influence model of patient-physician communication (sub-model: behavior, attitude, and relationship) Real-line arrows, gamma coefficients (*p*  < 0.05, power > 0.8); Dashed arrows, gamma coefficient (*p*  < 0.05, power < 0.8); Double-stranded arrows, psi coefficients

The four steps had significant progressive effects (β_12_ = 0.506, γ_23_ = 0.599, γ_34_ = 0.339, *p* < 0.05, power > 0.8), and patient education and counseling, in particular, had a significant additional effect on building a relationship (γ_24_ = 0.275, *p* < 0.05, power = 0.578 [< 0.8]). However, the power analysis was not sufficient (see Table 4). In order to investigate the reasons, we performed individual model analysis of the three components of the building a relationship step and found the following main differences: in the behavior relationship building model, both greeting and data gathering and patient education and counseling significantly and directly influenced behaviors (β^BR1^_14_ = 0.312, γ^BR1^_24_ = 0.480, *p* < 0.05, power > 0.8), but facilitation and patient activation had no effect on behaviors (γ^BR1^_34_ = -0.110, *p* > 0.05). However, in the attitudes and display of professionalism models, the path of influence was consistent with the overall model, with a significant single progressive influence (Figs. 1 and 2).

**Table 4:** Summary table of analytical coefficients affecting the overall relationship building path

**Effect Nature**	**Model Phase**	**DG, Greeting and Data Gathering**	**EC, Patient Education and Counseling**	**FA, Facilitation and Patient Activation**
**Direct Effect**	**EC, Patient Education and Counseling**	β_12_: 0.506 (0.102) [5.751*] {0.993}		
	**FA, Facilitation and Patient Activation**	β_13_: 0.133 (0.133) [1.518] {0.207}	γ_23_: 0.599 (0.115) [6.876*] {0.996}	
	**BR, Building a Relationship**	β_14_: 0.088 (0.112) [0.924] {0.000}	γ_24_: 0.275 (0.085) [3.089*] {0.578}	γ_34_: 0.339 (0.117) [2.402*] {0.815}
	**BR1,, Building a Relationship (Behavior)**	β^BR1^_14_: 0.312 (0.115) [3.342*] {0.873}	γ^BR1^_24_: 0.480 (0.120) [4.252*] {0.970}	γ^BR1^_34_: -0.110 (0.087) [-1.020] {0.012}
	**BR2, Building a Relationship (Attitude)**	β^BR2^_14_: 0.081 (0.120) [0.928] {0.000}	γ^BR2^_24_: 0.200 (0.125) [1.911] {0.477}	γ^BR2^_34_: 0.499 (0.091) [4.990*] {0.991}
	**BR3, Building a Relationship (Displaying Professionalism)**	β^BR3^_14_: -0.085 (0.145) [-0.800] {0.000}	γ^BR3^_24_: 0.115 (0.151) [0.886] {0.000}	γ^BR3^_34_: 0.395 (0.110) [3.186*] {0.843}
**Total Effect**	**BR (Overall)**	**0.375**	**0.478**	**0.339**
	**BR1 (Behavior)**	**0.507**	**0.414**	**-0.110**
	**BR2 (Attitude)**	**0.399**	**0.499**	**0.499**
	**BR3 (Displaying Professionalism)**	**0.145**	**0.352**	**0.395**

(): standard error, []:t test value, {}:statistical power, *: *p* < 0.05

This study performed a mean difference analysis to further explore the differences in patient-physician communication between the emergency department and other departments. As a consequence of prioritizing emergencies, the results showed that the emergency department performed significantly inferior in facilitation and patient activation, and building a relationship (F_FA_ = 10.685, *p* < 0.001, power = 0.999, η^2^ = 0.254; F_BR_ = 6.456, *p* < 0.01, power = 0.935, η^2^ = 0.151) (Table 5). The emergency department showed the lowest performance in facilitation and patient activation and a significantly lower performance in building a relationship than the family medicine and surgery departments. This suggests that the main differences in the process of patient-physician communication were due to the poorer performance of the emergency department in the facilitation and patient activation and building a relationship stage.

**Table 5:** Analysis of the differences between the four steps of communication and between active facilitation (FA) and relationship building (BR) in different disciplines

**Steps**	**Department**	**Number**	**Mean**	**Std. Deviation**	**Levene Statistic**	**F -test**	**Power**	**Effect size(Eta Squared)**	**Dunnett's T3**
DG, Greeting and Data Gathering	a Emergency	24	2.728	0.180	0.378	0.175	0.081	0.006	
	b Family medicine	24	2.718	0.210					
	c Internal	25	2.742	0.190					
	d Surgery	25	2.754	0.163					
EC, Patient Education and Counseling	a Emergency	24	2.688	0.250	1.472	2.152	0.533	0.064	
	b Family medicine	24	2.813	0.179					
	c Internal	25	2.800	0.172					
	d Surgery	25	2.820	0.224					
FA, Facilitation and Patient Activation	a Emergency	24	2.542	0.345	7.940***	10.685***	0.999	0.254	2,3,4 > 1
	b Family medicine	24	2.875	0.184					
	c Internal	25	2.885	0.191					
	d Surgery	25	2.840	0.233					
FA1 evaluation method	a Emergency	24	2.653	0.399	12.351***	4.264**	0.849	0.120	3 > 1
	b Family medicine	24	2.861	0.218					
	c Internal	25	2.920	0.174					
	d Surgery	25	2.827	0.257					
FA2 enhancement method	a Emergency	24	2.475	0.381	6.127***	11.699***	0.999	0.272	2,3,4 > 1
	b Family medicine	24	2.883	0.212					
	c Internal	25	2.864	0.250					
	d Surgery	25	2.848	0.254					
BR, Building a Relationship	a Emergency	24	2.718	0.308	6.456**	5.573**	0.935	0.151	2,4 > 1
	b Family medicine	24	2.917	0.118					
	c Internal	25	2.874	0.158					
	d Surgery	25	2.929	0.179					
BR1 Behavior	a Emergency	24	2.802	0.233	4.075**	2.135	0.529	0.064	
	b Family medicine	24	2.896	0.146					
	c Internal	25	2.790	0.295					
	d Surgery	25	2.920	0.187					
BR2 Attitude	a Emergency	24	2.688	0.359	7.638***	4.993**	0.904	0.137	2 > 1
	b Family medicine	24	2.924	0.147					
	c Internal	25	2.880	0.183					
	d Surgery	25	2.907	0.221					
BR3 Displaying Professionalism	a Emergency	24	2.861	0.339	4.151**	0.971	0.257	0.030	
	b Family medicine	24	2.903	0.269					
	c Internal	25	2.973	0.133					
	d Surgery	25	2.947	0.208					

^**^: *p* < 0.01; ***:*p* < 0.001

Analyzing these results of the communication factors in the emergency department involved in facilitation and patient activation and building a relationship yielded three significant findings. First, the internal department's performance in the evaluation method subtheme was significantly higher than that of the emergency department (F_FA1_ = 4.264, *p* < 0.01, power = 0.849, η^2^ = 0.120). Second, the performance of the family medicine, internal, and surgery departments in the enhancement method was significantly higher than that of the emergency department (F_FA2_ = 6.127, *p* < 0.001, power = 0.999, η^2^ = 0.272). Third, the performance of the family medicine department in attitudes was considerably higher than that of the emergency department (F_BR2_ = 4.993, *p* < 0.01, power = 0.904, η^2^ = 0.137).

## Discussion

Patient-physician communication in clinical settings in Taiwan could occur in four steps, starting from greeting and data gathering, then gradually moving into patient education and counseling and facilitation and patient activation to building a relationship. Each step involved two or three influencing factors from among the starting sentence, the contents inquired about, inquiry methods, topics in patient education and the health care system, description methods, evaluation methods, enhancement methods, behaviors, attitudes, and display of professionalism. The results of this study not only correspond to the RIAS model of communication [32–34] but also contribute 10 complementary and influential factors. This study also found that the four steps had significant progressive effects.

The results of this study showed that greeting and self-introduction before the doctor’s consultation, as influenced by Taiwan’s culture, can make a positive impression on the patient [12, 13] and that the attitude of listening and patience during the consultation [35] allows the patient to express feelings and fully confirm the reason for the consultation. This attitude can make the patient feel understood, which is essential for building the patient-physician relationship. It is an important element in establishing the patient-physician relationship and thus should be encouraged. Furthermore, while emphasizing the transfer of biomedical knowledge, physicians should pay attention to patients’ psychological perceptions and quality of life [16, 17] and use non-verbal behaviors or expressions, such as eye contact, gestures, and body movements [3, 18], which help to build successful relationships.

The patient-physician communication in the emergency department differs from that in other clinical settings and may be related to the fact that patients have only a few seconds of presentation time [4], which is a shorter time than that available to the average outpatient. Therefore, the first step in communicating causes the patient-physician relationship to be more stressful [8] and makes it more challenging to build positive and trusting relationships. This is especially true of emergency physicians who need to quickly identify symptoms and make medical decisions. On the other hand, the emergency department aims to provide short-term relief for acute illnesses; therefore, there is relatively little consideration of patients’ psychological feelings and active participation [5], with little emphasis on short-term or one-time relationship building. However, the patient’s perception of the relationship in the emergency department may be the basis for obtaining continued care in other specialties or for considering whether to transfer to another hospital [36]. Therefore, of the four communication steps considered in this study, the facilitation and patient activation step is integral to enhancing the attitude of emergency physicians and displaying their professionalism.

## Limitations

This study has several limitations. It used a quantitative questionnaire survey to examine patient-physician communication in four departments: internal, surgery, family medicine, and emergency department. Although 98 physicians participated in this study, the number of physicians from each department did not reach 30, which limits the inferences from the results. To mitigate this limitation, this study employed validation power in the analysis. It included effect size and the difference between the median and the overall median for each department to ensure the validity of the inference. However, surveys in future studies could involve larger samples. Another limitation of this study was that it was conducted in a rural, regional hospital; therefore, its results may not represent other types of hospitals. Therefore, clinical settings from various hospitals should be included and investigated in future studies.

## Conclusion

Good communication promotes trust and cooperation between patients and physicians. This study compared patient-physician communication between the emergency department and internal, surgery, and family medicine departments. The study has several findings. (a) The patient-physician communication model consisted of four steps and ten influencing factors. The steps included greeting and data gathering, patient education and counseling, facilitation, patient activation, and building a relationship. The factors were the starting sentence, the contents inquired about, inquiry methods, topics in patient education, description methods, evaluation method, enhancement method, behaviors, attitudes, and display of professionalism. (b) This study found that the four steps had significant progressive effects. Furthermore, good communication in the first step of greeting and data gathering will lead to positive performance in the next three steps. (c) The emergency department's performance in facilitating patient activation and building relationships was poor. The emergency department's performance in evaluation, enhancement, and attitude factors was significantly lower than that of other departments because emergency physicians focus mainly on biomedical issues and use closed-ended questions. To improve the performance of the emergency department in attitude and display of professionalism, physicians need to communicate well in the first three steps. (d) To establish a good relationship quickly, emergency physicians can begin with facilitation and patient activation to assess and encourage patients' activation to improve the quality of care.

Despite the high degree of uncertainty and complexity in the emergency department, communication systems can be enhanced through the collaborative efforts of the health care team, with nurse practitioners or multi-disciplinary professionals making up for the lack of time that physicians have to communicate effectively with their patients and working together to build rapport. The results of this study can be used as a reference for medical and patient communication education and training programs to strengthen behavioral performance in the four stages in a gradual, step-by-step manner. In particular, emergency departments need to maintain their performance in facilitation and patient activation to assess and encourage patients’ positivity, establish a good relationship quickly, and improve the quality of medical care and commitment. The aim is to establish a good relationship speedily and enhance the quality of care without jeopardizing attitudes or professional performance. We recommend installing an effective communication and cooperation model based on teamwork for the emergency department. Training can be provided, and effectiveness can be evaluated according to the stages of patient-physician communication considered in this study to improve communication skills, which can serve as the basis for empirical research on communication between patients and medical professionals.

### Supplementary Information

**Additional file 1. **"Interview Outline of Patient-Physician Communication Behavior" was used as a tool for the qualitative data collection.

**Additional file 2. **"Survey of Patient-Physician Communication Behavior" was used as a tool for the quantitative data collection.

## Data Availability

The datasets used and/or analyzed in the present study are available from the corresponding author upon reasonable request.
