# Supplementary material for: Differences in patient-physician communication between the emergency department and other departments in a hospital setting in Taiwan
Source: BMC Health Serv Res. 2023 Nov 20;23:1279. doi: 10.1186/s12913-023-10311-2 (PMC10662510; doi:10.1186/s12913-023-10311-2)
Supplement: Supplementary file 1 — Additional file 1. "Interview Outline of Patient-Physician Communication Behavior" was used as a tool for the qualitative data collection. [file 12913_2023_10311_MOESM1_ESM.docx]

**Interview Outline of Patient-Physician Communication Behavior**

- Personal Information

1. Gender:
2. Age:
3. Medical Specialty:
4. Years of experience as an attending physician:

- Patient-Physician Communication Behavior

1. How are patients interviewed to gather information about their condition?
2. How are patients asked to collect information about the disease? How is the disease and health counseling explained to patients?
3. How are good relationships established with patients?
4. How is patients' compliance with medical advice assessed? How are the ways to improve patients' compliance assessed?
