# Supplementary material for: Differences in patient-physician communication between the emergency department and other departments in a hospital setting in Taiwan
Source: BMC Health Serv Res. 2023 Nov 20;23:1279. doi: 10.1186/s12913-023-10311-2 (PMC10662510; doi:10.1186/s12913-023-10311-2)
Supplement: Supplementary file 2 — Additional file 2. "Survey of Patient-Physician Communication Behavior" was used as a tool for the quantitative data collection. [file 12913_2023_10311_MOESM2_ESM.docx]

**Survey of Patient-Physician Communication Behavior**

- Personal Information

1. Gender: □ Male □ Female
2. Age: __________
3. Medical Specialty: □ Internal Medicine □ Surgery □ Family Medicine □ Emergency Department
4. Years of experience as an attending physician: □ Less than one year of experience □ One to three years of experience □ Four to six years of experience □ Seven to nine years of experience □ Ten or more years of experience

- Patient-Physician Communication Behavior

Please select the communication behaviors that you have actually used in the process of communication with patients. Check "Generally have" for any behaviors you have used, regardless of how often. Check "Generally do not" for behaviors that you have not used in your interactions with patients.

| **Factors** | **Communication Behaviors** | **Generally have** | **Depends on the situation** | **Generally do not** |
| --- | --- | --- | --- | --- |
| Greeting and Data Gathering | 1. I begin by greeting the patient and introducing myself. |  |  |  |
|  | 1. I first confirm the patient's identity, such as asking, "Is this Mr. Zhang?" |  |  |  |
|  | 1. I confirm the language the patient uses or understands. |  |  |  |
|  | 1. I first observe the patient's condition, such as gait, posture, or facial expressions, and provide appropriate inquiries and care. |  |  |  |
|  | 1. I inquire about the patient's main complaints. |  |  |  |
|  | 1. I will extend inquiries based on the patient's main complaints. |  |  |  |
|  | 1. I inquire about the patient's personal information and medical history. |  |  |  |
|  | 1. I inquire and confirm the relationship between the patient and the accompanying individuals. |  |  |  |
|  | 1. I guide the patient to state the reasons for the visit and any concerns. |  |  |  |
|  | 1. I use closed-ended questions for inquiries. |  |  |  |
|  | 1. I provide options for the patient to choose from to clarify their issues. |  |  |  |
|  | 1. I use written words or illustrations to help with enquiring. |  |  |  |
|  | 1. I ask accompanying caregivers or family members to assist in explaining the condition. |  |  |  |
|  | 1. Other behaviors: | | | |
| Patient Education and Counseling | 1. I explain the patient the symptoms and the subsequent medical procedures. |  |  |  |
|  | 1. I provide explanations for the questions or concerns the patient may have. |  |  |  |
|  | 1. I explain the pathological and clinical statistical data. |  |  |  |
|  | 1. I explain the medication prescriptions and precautions. |  |  |  |
|  | 1. I provide caregivers or family members information of proper home care. |  |  |  |
|  | 1. I inquire and confirm the patient's understanding of the health education. |  |  |  |
|  | 1. I use online resources or images for health education. |  |  |  |
|  | 1. I use everyday metaphors for explanations. |  |  |  |
|  | 1. I provide explanations using words that the patient can comprehend. |  |  |  |
|  | 1. I adjust the manner and content of explanations based on the patient's education level and occupation. |  |  |  |
|  | 1. I adapt my speech speed and volume according to the patient's age and conditions. |  |  |  |
|  | 1. I ask caregivers or family members to assist in explaining medical instructions. |  |  |  |
|  | 1. Other behaviors: | | | |
| Facilitation and Patient Activation | 1. I assess the patient's compliance with medical prescriptions based on their conditions and test results. |  |  |  |
|  | 1. I inquire about the patient's medication adherence to understand their compliance with medical prescriptions. |  |  |  |
|  | 1. I ask caregivers or family members about their compliance with medical prescriptions. |  |  |  |
|  | 1. I understand the reasons why the patient has not adhered to medical prescription. |  |  |  |
|  | 1. I collaborate with healthcare professionals and family members to assist the patient in following medical prescriptions. |  |  |  |
|  | 1. I communicate with caregivers or family members to reach a consensus on compliance with medical prescriptions. |  |  |  |
|  | 1. For patients who have not followed medical prescriptions, I will discuss solutions with them. |  |  |  |
|  | 1. I encourage the patient to actively participate in medical treatments and comply with medical prescriptions. |  |  |  |
|  | 1. Other behaviors: | | | |
| Building a Relationship | 1. When speaking to the patient, I use body language or gestures. |  |  |  |
|  | 1. During examinations, I will listen to the patient's speech. |  |  |  |
|  | 1. I provide the patient with sufficient time to express their opinions and thoughts. |  |  |  |
|  | 1. During examinations, I ensure eye contact with the patient. |  |  |  |
|  | 1. I proactively inquire about the patient's conditions and provide psychological support. |  |  |  |
|  | 1. I will be patient and repeatedly confirm issues with the patient. |  |  |  |
|  | 1. Both the patient and I express our opinions and thoughts fully. |  |  |  |
|  | 1. I can reach a consensus with the patient on medical treatments. |  |  |  |
|  | 1. There is mutual trust between the patient and me. |  |  |  |
|  | 1. I demonstrate high willingness to assist the patient in problem-solving. |  |  |  |
|  | 1. I understand and clarify the patient's needs for consultations and medical treatments. |  |  |  |
|  | 1. I assist the patient in reducing their symptoms. |  |  |  |
|  | 1. I provide options of medical treatments and offer recommendations. |  |  |  |
|  | 1. Other behaviors: | | | |
